# Supplementary material for: Sulfate triple-oxygen-isotope evidence confirming oceanic oxygenation 570 million years ago
Source: Nat Commun. 2023 Jul 18;14:4315. doi: 10.1038/s41467-023-39962-9 (PMC10354052; doi:10.1038/s41467-023-39962-9)
Supplement: Supplementary file 1 — Supplementary Information [file 41467_2023_39962_MOESM1_ESM.pdf]

Supplementary Information for

Sulfate triple-oxygen-isotope evidence confirming oceanic oxygenation 570 million years ago

Haiyang Wang[1,2,3], Yongbo Peng[1,4]*, Chao Li[2,3,5]*, Xiaobin Cao[1,4], Meng Cheng[2,3,5], Huiming Bao[1,4]*

[1]*International Center for Isotope Effects Research, Nanjing University, Nanjing 210023, China*

[2]*State Key Laboratory of Oil and Gas Reservoir Geology and Exploitation & Institute of Sedimentary Geology, Chengdu University of Technology, Chengdu 610059, China*

[3]*International Center for Sedimentary Geochemistry and Biogeochemistry Research, Chengdu University of Technology, Chengdu 610059, China*

[4]*Frontiers Science Center for Critical Earth Material Cycling and State Key Laboratory for Mineral Deposits Research, School of Earth Sciences and Engineering, Nanjing University, Nanjing 210023, China*

[5]*Key Laboratory of Deep-time Geography and Environment Reconstruction and Applications of Ministry of Natural Resources, Chengdu University of Technology, Chengdu 610059, China*

**Corresponding authors. Email: ybpeng@nju.edu.cn (Y. P.); chaoli@cdut.edu.cn (C. L.); bao@nju.edu.cn (H. B.).*

This PDF file includes:

Supplementary Discussion

Supplementary Figures 1 to 7

Supplementary Tables 1 to 3

Supplementary References

Supplementary Discussion

Correlation of Shuram Excursion in different paleocontinents.

The long-duration, negative $\delta^{13}C_{carb}$ excursions in the Doushantuo (South China) and Wonoka (South Australia) formations are widely believed to be equivalent to the Shuram Excursion (SE)[1–4]. The negative $\delta^{13}C_{carb}$ excursion with a nadir of −11.9‰ recorded in the Shuiquan Formation in Tarim should also be correlated with the Shuram excursion, in light of the following lines of evidence.

First, U-Pb ages have placed the Shuiquan Formation to be between 615±6 Ma and 541±6 Ma[5,6], which supports the Ediacaran age of the succession; Vendotaenid fossils have been reported from the Shuiquan Formation, which are commonly found in post-Marinoan rocks[7]. Second, no other long-duration, negative $\delta^{13}C_{carb}$ excursion (as low as −12‰) has been reported between 615 Ma and 541 Ma on any continent globally, except for the SE or the equivalent Wonoka/DOUNCE/EN3[1–4]. Third, the negative $\delta^{13}C$ excursion at Mochia-Khutuk section in NE Tarim is comparable in magnitude, pattern of shift, lithology, and sedimentology to the largest $\delta^{13}C$ anomaly (lasting for >50 m with a nadir at ca. −12‰) observed in Aksu area, NW Tarim, which has been correlated with the SE based on multiple lines of evidence and precise age constraints[8]. Fourth, other unique geochemical signals, e.g., the decoupling of $\delta^{13}C_{carb}$ and $\delta^{13}C_{org}$ and a concurrent decrease in $\delta^{18}O$ values, as commonly observed in other typical SE sections, were found in the Mochia-Khutuk section (Supplementary Data 1). Fifth, the Mochia-Khutuk section has a "M-shape" pattern in the concentration of carbonate-associated phosphate[9], a feature observed in global SE sections including the Jiulongwan (the same section as reported in our study) and Sishang sections of the Doushantuo Formation from South China, the Cerro Rajón section of the Clemente, Pitiquito and Gamuza formations from northern Mexico, the Death Valley section of

the Johnnie Formation from southwestern USA, and the Parachilna Gorge section of the Wonoka Formation (the same section as reported in our study) from South Australia[9].

Sulfur cycle modeling

The model considers both sulfate fluxes and changes in sulfate isotope composition over time, allowing $\delta^{34}S$ and $\delta^{18}O$ to be altered by microbial sulfur redox processes as prescribed in previous models[10,11]. For the $\Delta'^{17}O$, we assume the newly added sulfate from pyrite weathering on the continents and from aerobic oxidation of reduced sulfur species (e.g., H_2S and S^0) in the ocean carry negative ^{17}O anomalies. The ^{17}O anomaly is erased by sulfur redox processes through enhanced exchange of sulfate oxygen with the ambient water[12,13]. All of these processes have been taken into consideration in the modeling.

1. Model description

The marine sulfur cycle is governed by the following mass (flux) conservation equation:

$$\frac{d}{dt}M_{\mathrm{i\ SO4}}(t)=F_{\mathrm{i\ wp}}(t)+F_{\mathrm{i\ we}}(t)+F_{\mathrm{i\ v}}(t)-F_{\mathrm{i\ be}} - F_{\mathrm{i\ MSR}}(t)+F_{\mathrm{i\ MSR\text{-}r}}(t)+F_{\mathrm{i\ reo\text{-}aerobic}}(t)+F_{\mathrm{i\ reo\text{-}anaerobic}}(t) + F_{\mathrm{i\ extra}} \qquad (1)$$

where *i* denotes mass of 16, 17, 18, 32 or 34, $M_{\mathrm{i\ SO4}}$ denotes the mass of each isotope in marine sulfate pool, $F_{\mathrm{i\ wp}}$, $F_{\mathrm{i\ we}}$, and $F_{\mathrm{i\ v}}$ denote the fluxes of each sulfur isotope from pyrite weathering, evaporite dissolution, and mantle degassing, respectively. $F_{\mathrm{i\ be}}$, $F_{\mathrm{i\ MSR}}$, and $F_{\mathrm{i\ MSR\text{-}r}}$ denote the flux of each isotope from evaporite burial, the gross forward and intracellular backward fluxes of each isotope during the microbial sulfate reduction (MSR), respectively. $F_{\mathrm{i\ reo\text{-}aerobic}}$ and $F_{\mathrm{i\ reo\text{-}anaerobic}}$ denote fluxes of each isotope during

aerobic and anaerobic sulfur oxidation respectively. $F_{i\ extra}$ denotes each isotope flux of a potentially added sulfate source from aerobic oxidation of a pre-existing sulfur (H_2S/S^0) reservoir, and all fluxes are in mol yr^{-1}.

We assume the total masses of S and O equal to sums of the respective major isotopes:

$$S = {}^{32}S + {}^{34}S \qquad (2)$$

$$O = {}^{16}O + {}^{18}O \qquad (3)$$

where ${}^{32}S$, ${}^{34}S$, ${}^{16}O$, and ${}^{18}O$ denote the masses of the respective isotope.

The individual mass for each isotope thus can be calculated by the respective δ values with S and O masses:

$${}^{32}S = \frac{S \times 1000}{\mathrm{R_{VCDT}}(\delta^{34}\mathrm{S} + 1000) + 1000} \qquad (4)$$

$${}^{34}S = \frac{\mathrm{R_{VCDT}}(\delta^{34}\mathrm{S} + 1000)S}{\mathrm{R_{VCDT}}(\delta^{34}\mathrm{S} + 1000) + 1000} \qquad (5)$$

$${}^{16}O = \frac{O \times 1000}{\mathrm{R_{VSMOW18}}(\delta^{18}\mathrm{O} + 1000) + 1000} \qquad (6)$$

$${}^{17}O = \frac{\mathrm{R_{VSMOW17}}(\delta^{17}\mathrm{O} + 1000)O}{\mathrm{R_{VSMOW18}}(\delta^{18}\mathrm{O} + 1000) + 1000} \qquad (7)$$

$${}^{18}O = \frac{\mathrm{R_{VSMOW18}}(\delta^{18}\mathrm{O} + 1000)O}{\mathrm{R_{VSMOW18}}(\delta^{18}\mathrm{O} + 1000) + 1000} \qquad (8)$$

where R_{VCDT} and R_{VSMOW} denote the absolute isotopic ratios of standard materials for VCDT and for VSMOW, respectively.

Since oxygen isotope fractionation during evaporite dissolution, mantle degassing, MSR, and anaerobic oxidation of sulfur are mass-dependent, we calculate their $\delta^{17}O$ using a diagnostic exponent value of 0.528 with $\delta^{18}O$:

$$\delta^{17}\mathrm{O} = ((\delta^{18}\mathrm{O}/1000 + 1)^{0.528} - 1) \times 1000 \qquad (9)$$

The triple oxygen isotope exponent (${}^{17}\theta_{msr}$) of the MSR was suggested to be ~0.528[14,15],

but here we consider a wider range of $^{17}\theta_{msr}$ from 0.520 to 0.530 in the model.

For sulfate pools that bear ^{17}O depletion, i.e., sulfate sourced from pyrite oxidative weathering on land or aerobic oxidation of sulfur in seawater ($\Delta'^{17}O_{sul\text{-}oxidized}$), we calculate their $\delta^{17}O$ as below:

$$\delta^{17}\mathrm{O} = (\exp(\Delta'^{17}\mathrm{O}/1000 + 0.5305 \times \ln(\delta^{18}\mathrm{O}/1000 + 1)) - 1) \times 1000 \qquad (10)$$

The pyrite burial flux (F_{bp}) was explored as a function of seawater sulfate concentration using a reaction-transport model within sediments under modern conditions[16] as below:

$$F_{\mathrm{bp}} = \frac{0.962 \times 10^{12} \times [\mathrm{SO_4}](\mathrm{t})}{1.8685 + [\mathrm{SO_4}](\mathrm{t})} \qquad (11)$$

where $[SO_4](t)$ denotes seawater sulfate concentration in mM.

As the fraction of H_2S-reoxidized (f_{reo}) during MSR is suggested to be 0.8–0.95 in the modern oceans[17,18], we convert F_{bp} to the net sulfate reduction flux (f_{MSR}) with f_{reo} fixed at 0.88:

$$f_{MSR} = \frac{F_{\mathrm{bp}}}{1 - f_{\mathrm{reo}}} = \frac{8.017 \times 10^{12} \times [\mathrm{SO_4}](\mathrm{t})}{1.8685 + [\mathrm{SO_4}](\mathrm{t})} \qquad (12)$$

A sulfate concentration of 28 mM thereby yields a modern ocean sulfate reduction rate of 7.52×10^{12} mol yr^{-1}, almost identical to the estimated value (7.7×10^{12} mol yr^{-1}) based on the continental shelf area and sulfate reduction rate[19].

The gross forward F_{MSR} is then calculated as:

$$F_{\mathrm{MSR}} = \frac{f_{\mathrm{msr}}}{1 - r_{\mathrm{msr}}} = \frac{8.017 \times 10^{12} \times [\mathrm{SO_4}](\mathrm{t})}{(1.8685 + [\mathrm{SO_4}](\mathrm{t}))(1 - r_{\mathrm{msr}})} \qquad (13)$$

where r_{msr} denotes the ratio of the intracellular backward to forward flux during MSR.

We calculated r_{msr} based on the ratio of the apparent S-isotope fractionation ($\alpha^{34}S_{MSR}$) to the complete expression of 70‰ during the MSR[20]:

$$r_{\mathrm{msr}} = \frac{\alpha^{34}\mathrm{S_{MSR}} - 1}{70‰} \quad (14)$$

Note that the r_{msr} value was kept constant when testing varied $\alpha^{34}S_{MSR}$ and r_{msr} values for different cases.

If MSR processes have occurred extensively in the anoxic water-column rather than only within the sediments, as during the Paleocene-Eocene Thermal Maximum, the gross MSR flux could be one order of magnitude higher than modern ocean where MSR occurs mainly within the sediments[10]. It is necessary to take this factor into account especially for the Precambrian oceans. Therefore, we added a variable coefficient (k_{msr}) for MSR flux in respond to changes of water-column redox conditions, and obtain Eqs. (15) and (16):

$$F_{\mathrm{MSR}} = (1 + k_{\mathrm{msr}}) \times \frac{8.017 \times 10^{12} \times [\mathrm{SO_4}](\mathrm{t})}{(1.8685 + [\mathrm{SO_4}](\mathrm{t}))(1 - r_{\mathrm{msr}})} \quad (15)$$

and

$$F_{\mathrm{MSR-r}} = (1 + k_{\mathrm{msr}}) \times \frac{8.017 \times 10^{12} \times [\mathrm{SO_4}](\mathrm{t}) \times r_{\mathrm{msr}}}{(1.8685 + [\mathrm{SO_4}](\mathrm{t}))(1 - r_{\mathrm{msr}})} \quad (16)$$

where k_{msr} at 0 represents the case when the water-column is well-oxygenated, with $[O_2]$ higher than ~4 µM for most water masses. 4 µM appears to be the threshold $[O_2]$ below which the MSR will replace oxic respiration as the main remineralization pathway of organic matter[21].

The $F_{reo\text{-}aerobic}$ was calculated with a variable fraction ($f_{reo\text{-}aerobic}$), namely the proportion of sulfate from aerobic oxidation of H_2S/S^0 among all sulfate fluxes from the re-oxidation of reduced H_2S/S^0 species produced during the course of MSR:

$$F_{\mathrm{reo-aerobic}} = f_{\mathrm{reo-aerobic}} \times f_{\mathrm{MSR}} \times f_{\mathrm{reo}} \quad (17)$$

and the $F_{reo\text{-}anaerobic}$, the fraction of anaerobic sulfur reoxidation, is obtained by:

$$F_{\mathrm{reo-anaerobic}} = (1 - f_{\mathrm{reo-aerobic}}) \times f_{\mathrm{MSR}} \times f_{\mathrm{reo}} \quad (18)$$

The F_{be} is set as a linear function of seawater sulfate concentration, which is assumed as:

$$F_{be} = \frac{[SO_4](t) \times 1.12 \times 10^{12}}{28} \qquad (19)$$

where $[SO_4](t)$ denotes seawater sulfate concentration in mM, and 1.12×10^{12} mol yr^{-1} is the suggested evaporite burial flux at a steady-state modern ocean[22].

2. Parameter constraints

(1) The initial fluxes of evaporite and pyrite weathering, and the mantle degassing, as well as their sulfate S- and O-isotope compositions are listed in Supplementary Table 2. All parameters, except for the flux of pyrite weathering, remain unchanged during the course of model runs.

(2) The isotope composition of buried evaporite is equal to that of seawater sulfate, which changes through time.

(3) The $\delta^{34}S$ of the sulfate derived from sulfide oxidation inherits that of the sulfide.

(4) During the course of MSR, part of the sulfate that is reduced can be subsequently re-oxidized to sulfate via three main pathways: (i) the intracellular sulfite -sulfate exchange equilibrium catalysed by enzymes[12], (ii) anaerobic oxidation of sulfur/sulfide by NO_3^-, Fe(III), etc[13]. (iii) aerobic oxidation of sulfur/sulfide by O_2[23,24]. Note that the sulfate from 'i' and 'ii' pathways do not carry ^{17}O anomalies, because all of newly-formed sulfate oxygen atoms are derived from the ambient water, whereas sulfate from pathway 'iii' have the potential to inherit negative ^{17}O anomalies from the dissolved O_2. The $\delta^{18}O$ of the intracellular sulfate derived from sulfite is set to 25‰ based on the equilibrium fractionation factor between sulfate and water (~ 23-29‰)[25–27]. The $\delta^{18}O$ of sulfate from anaerobic oxidation of sulfides is set to 21‰, as sulfur disproportionation plays an important role in sulfate formation[28]. We use 0-8‰ as the

$\delta^{18}O$ value of the sulfate derived from aerobic oxidation of sulfur/sulfide[29–31]. There are uncertainties in selecting the respective $\delta^{18}O$ values for these three end-member sulfate pools, which will impact the absolute values of parameters, such as f_{reo} and $f_{reo\text{-}aerobic}$, i.e., the relative fractions from different sulfate sources. However, these uncertainties will have a minimal impact on the mechanisms and processes we explored here, as our discussion and conclusions are developed based on the relative changes in these variables rather than their absolute values.

3. Steady-state model initialization

We used the South China dataset as a typical case, scaled the variables and constructed multiple scenarios of initial steady state by fitting the $\delta^{34}S$ at ~40‰, $\delta^{18}O$ at ~20‰, and $\Delta'^{17}O$ at ~ −0.17‰ before the SE (Supplementary Table 3). The establishment of these steady states requires either MSR processes occurring mainly in the water-column ($k_{msr} > 0$), or the contribution of a fraction of sulfate from H_2S-reoxidation (f_{reo} 0.5-0.9) and a fraction from aerobic sulfur oxidation ($f_{reo\text{-}aerobic}$ 0-0.3). The corresponding sulfate concentration ranges from 0.2 to 6.0 mM, which is mainly controlled by MSR (k_{msr}) and the fluxes of sulfate from sulphide/sulfur reoxidation (f_{reo}). The $\delta^{34}S$ of the produced H_2S/pyrite ranges from ~0.3 to 3‰ during MSR, and the associated sulfur isotope offset is at the range of ~37 to 40‰, similar to estimates from other sulfur cycle models[22,32,33]. The r_{msr} is estimated to be at 0.54-0.58 based on the $\alpha^{34}S_{MSR}$ values of 0.961-0.964. The $^{17}\theta_{msr}$ of 0.52-0.53 corresponds to seawater sulfate $\Delta'^{17}O$ from −0.03 to −0.17‰ if assuming the $\Delta'^{17}O_{sul\text{-}oxidized}$ to be 0‰. The average $\Delta'^{17}O$ of −0.17‰ observed in the pre-SE HCl-leached CAS samples is met when the $\Delta'^{17}O_{sul\text{-}oxidized}$ ranges from −0.75 to −0.85‰ ($^{17}\theta_{msr}$ = 0.525-0.528).

4. Sensitivity analyses and model runs

We used the initial steady states SS10-2 and SS11-2 (Supplementary Table 3) to

do sensitivity analyses for F_{wp}, k_{msr}, f_{reo}, and $f_{reo\text{-}aerobic}$, all of which are linked to the supply of sulfate with negative $\Delta'^{17}O$ values (Supplementary Fig. 4). Results showed that F_{wp} ($\delta^{34}S_{py}$ = −17‰), k_{msr} and f_{reo} have a larger effect on the $\delta^{34}S$ than on the $\delta^{18}O$, while $f_{reo\text{-}aerobic}$ only influences the $\delta^{18}O$. None of them can simultaneously reproduce the observed $\delta^{34}S$ (~20‰) and $\delta^{18}O$ (~12‰) values during the SE. Instead, the F_{wp} ($\delta^{34}S_{py}$ = 18‰) can match both the $\delta^{34}S$ and $\delta^{18}O$ shifts well, which, however, requires an unrealistic increase in the F_{wp}, corresponding to an unrealistic increase in marine sulfate concentration (reaching up to ~300 mM).

To explore the uncertainty in the choice of initial steady-state sulfate concentration, we ran SS14-2 ([SO_4]: 6.0 mM) exactly the same as the initial steady state SS4-2 and SS8-2 for comparison (Supplementary Fig. 5). It can reproduce the observed S- and O-isotope changes as well, and the required sulfate flux is the same as the scenario SS4-2 and SS8-2. This result indicates that the choice of the initial steady-state sulfate concentration is insensitive to the modeled sulfate fluxes during the course of the SE and has no influence on the mechanisms affecting the isotope composition we explored.

Continuing on the basis of the SS14-2 case, we changed $^{17}\theta_{msr}$ from 0.525 to 0.52 or 0.53 (Supplementary Fig. 6). Results showed that the modelled sulfate $\Delta'^{17}O$ during the SE is not sensitive to the change of $^{17}\theta_{msr}$. Thus, our estimated $\Delta'^{17}O$ value from −0.7 to −0.8‰ for the newly-formed sulfate is not affected by the uncertainty in the $^{17}\theta_{msr}$. We also changed the isotope composition of the sulfate supplied from oxidation of the pre-existing sulfur pool, to explore other fluxes that are required to reproduce the observed sulfur and oxygen isotopes during the SE (using SS8-2; Supplementary Fig. 7). The results showed that the required sulfate flux from the sulfur oxidation could reach up to 1.0-6.0 $\times 10^{13}$ mol yr^{-1}.

Supplementary Figures

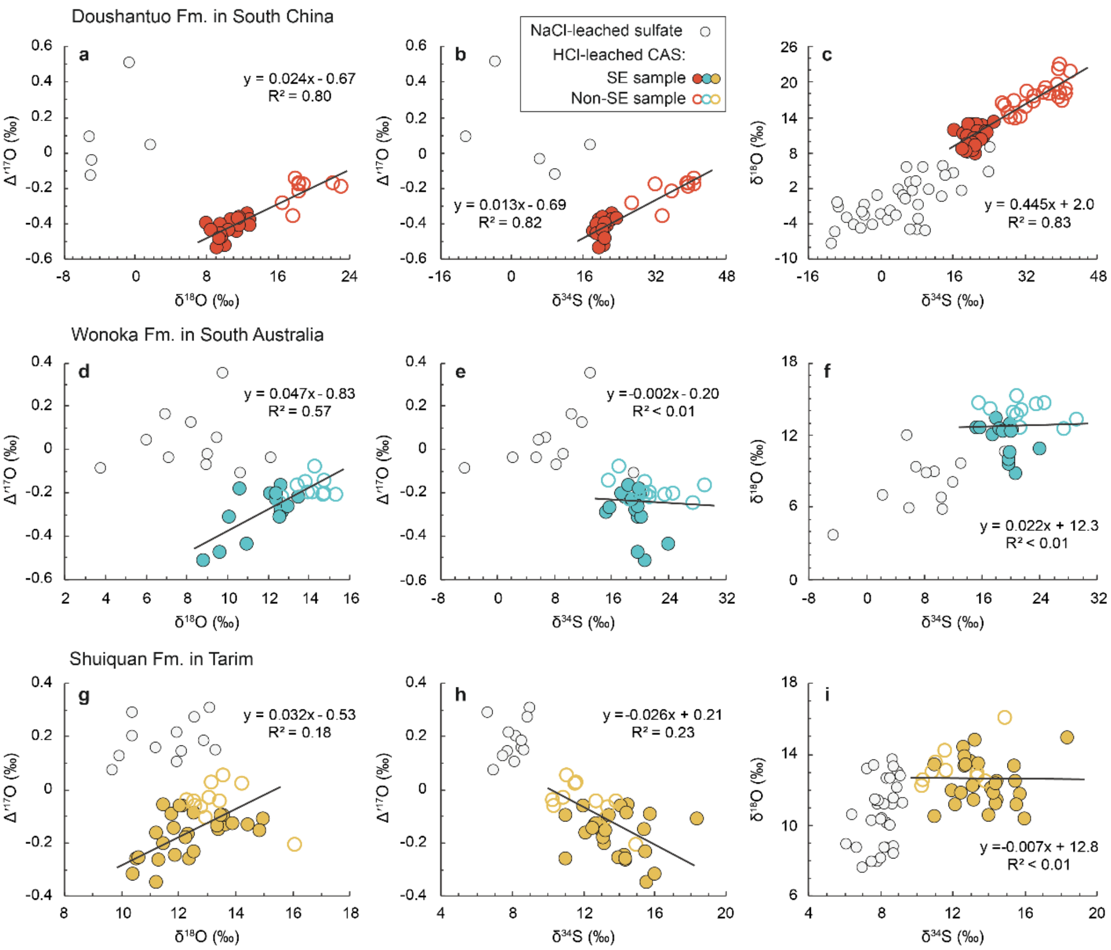

Supplementary Fig. 1. Crossplots of Δ'^{17}O, δ^{18}O and δ^{34}S in the HCl-leached and the NaCl-leached sulfate. Data from the Doushantuo Fm. in South China (**a-c**), the Wonoka Fm. in South Australia (**d-f**), and the Shuiquan Fm. in Tarim (**g-i**). Strong positive correlations are observed among $\Delta'^{17}O_{CAS}$, $\delta^{18}O_{CAS}$ and $\delta^{34}S_{CAS}$ for data from the Doushantuo Fm. (**a-c**). The Wonoka Fm. exhibits a good positive correlation between $\Delta'^{17}O_{CAS}$ and $\delta^{18}O_{CAS}$ (**d**), but not between $\Delta'^{17}O_{CAS}$ and $\delta^{34}S_{CAS}$ (**e**) or between $\delta^{34}S_{CAS}$ and $\delta^{18}O_{CAS}$ (**f**). The Shuiquan Fm. shows a weak positive correlation between $\Delta'^{17}O_{CAS}$ and $\delta^{18}O_{CAS}$ (**g**), a weak negative correlation between $\Delta'^{17}O_{CAS}$ and $\delta^{34}S_{CAS}$ (**h**), and no relationship between $\delta^{34}S_{CAS}$ and $\delta^{18}O_{CAS}$ (**i**).

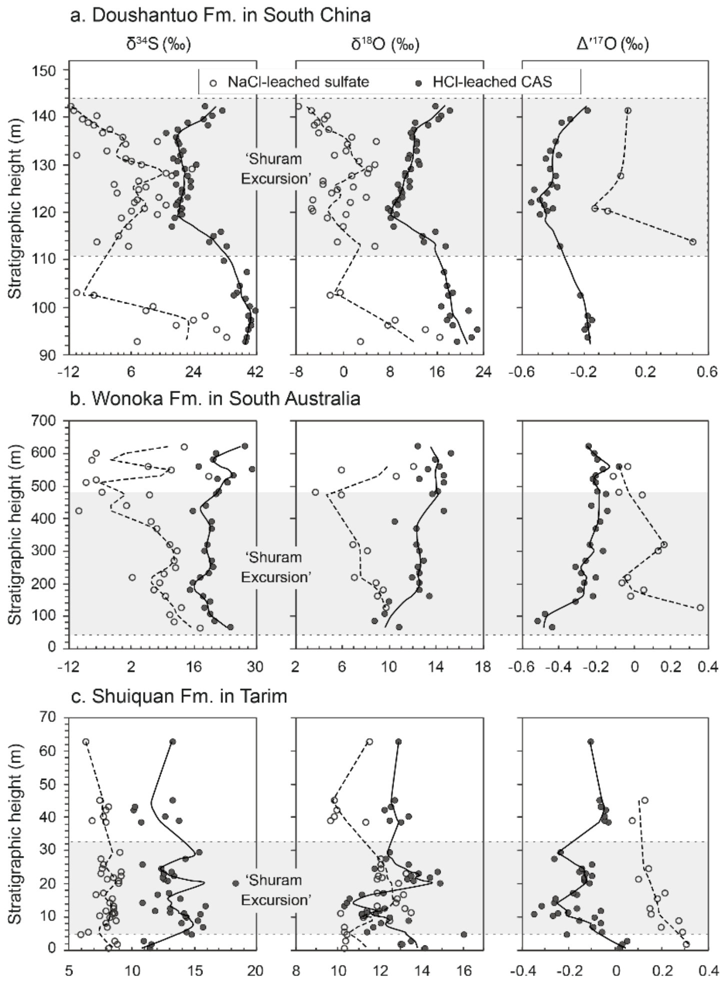

Supplementary Fig. 2. Sulfur and oxygen isotope compositions of the NaCl-leached and the HCl-leached sulfate of the Ediacaran successions from South China (**a**), South Australia (**b**) and Tarim (**c**). The shaded areas refer to intervals of the largest negative carbon isotope excursion (defined here by $\delta^{13}C_{carb}$ lower than −6‰). The solid and dash lines in each column represent LOWESS curves.

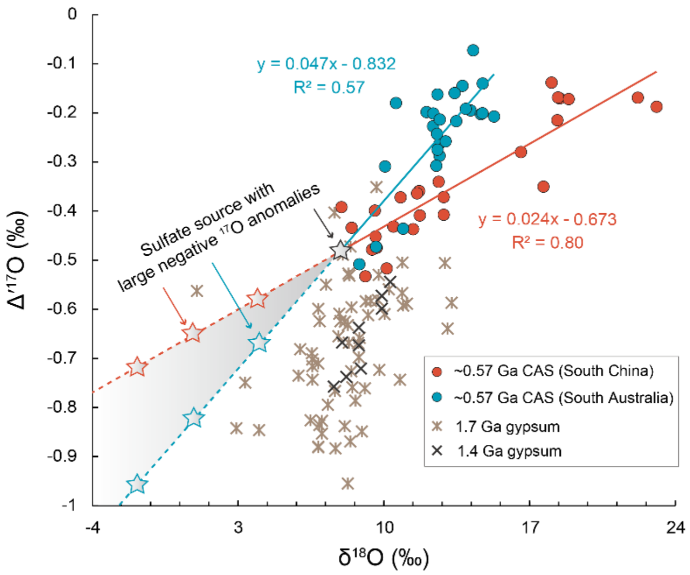

Supplementary Fig. 3. Crossplots of the $\Delta'^{17}O$ and $\delta^{18}O$ for Ediacaran CAS and for the pre-Ediacaran gypsum with significantly negative ^{17}O anomalies. Strong positive correlations between $\Delta'^{17}O_{CAS}$ and $\delta^{18}O_{CAS}$ in South China and South Australia suggest a mixture between two end-member sulfate pools, one with a higher $\delta^{18}O$ and a close-to-zero $\Delta'^{17}O$ value, and the other with a lower $\delta^{18}O$ and a negative $\Delta'^{17}O$ value. The sulfate source associated with the latter pool, represented by the five-pointed stars, can be estimated based on the correlation trends of $\Delta'^{17}O$ and $\delta^{18}O$. Notably, the data for pre-existing (i.e., 1.4-Ga and 1.7-Ga) gypsum with significantly negative ^{17}O anomalies[34,35] do not align with the region defined by the red and blue dashed lines as well as the surrounding grey shaded area, which represents isotopic composition of the potential end-member sulfate. Therefore, it does not support pre-Ediacaran gypsum as one of the end-member sulfate pools.

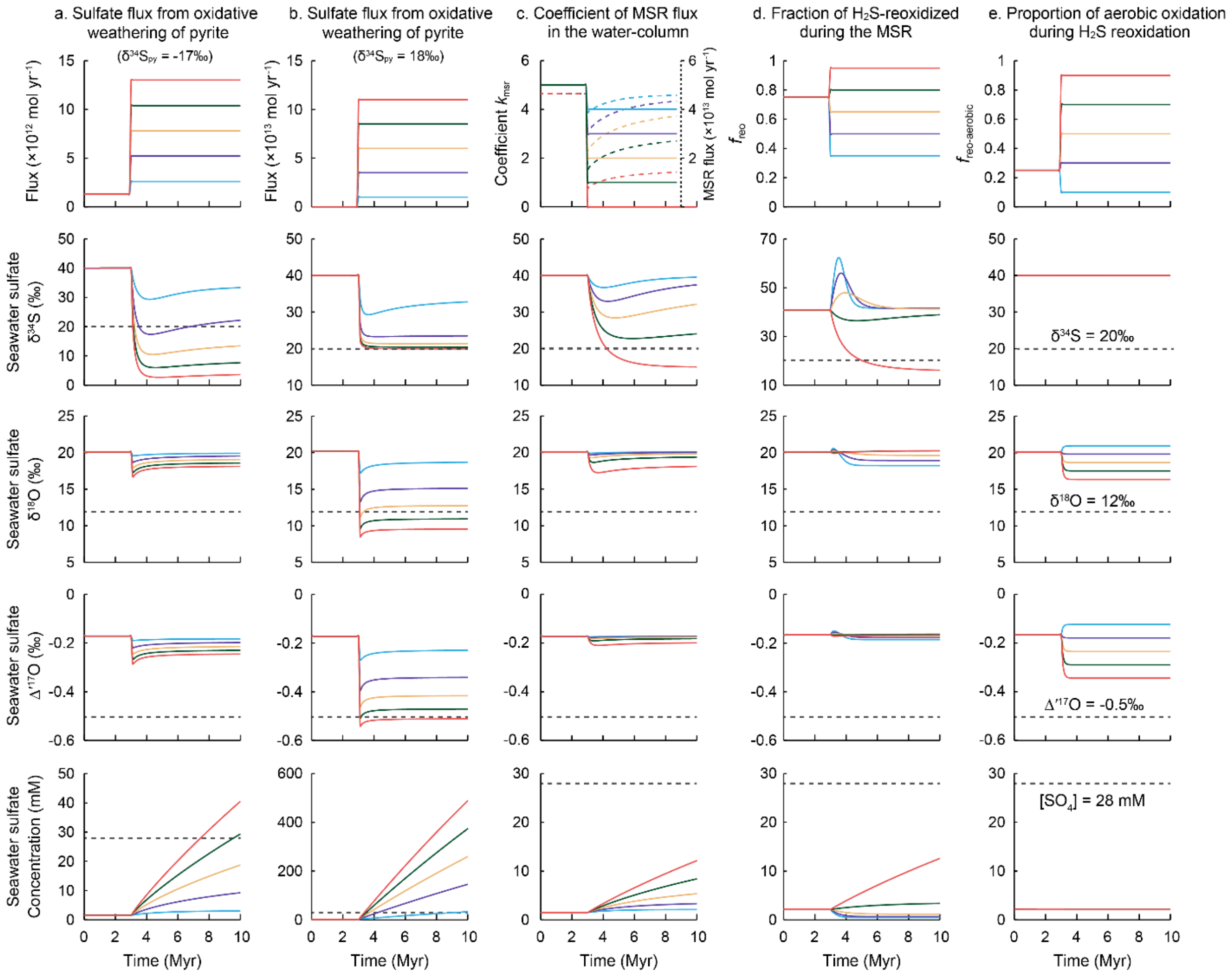

Supplementary Fig. 4. Sensitivity tests of sulfur and oxygen isotope composition of sulfate on pyrite weathering flux (F_{wp}) (**a-b**), coefficient of MSR flux in the water-column (k_{msr}) (**c**), fraction of H_2S-reoxidized during the MSR (f_{reo}) (**d**), and proportion of aerobic oxidation during H_2S reoxidation ($f_{reo\text{-}aerobic}$) (**e**).

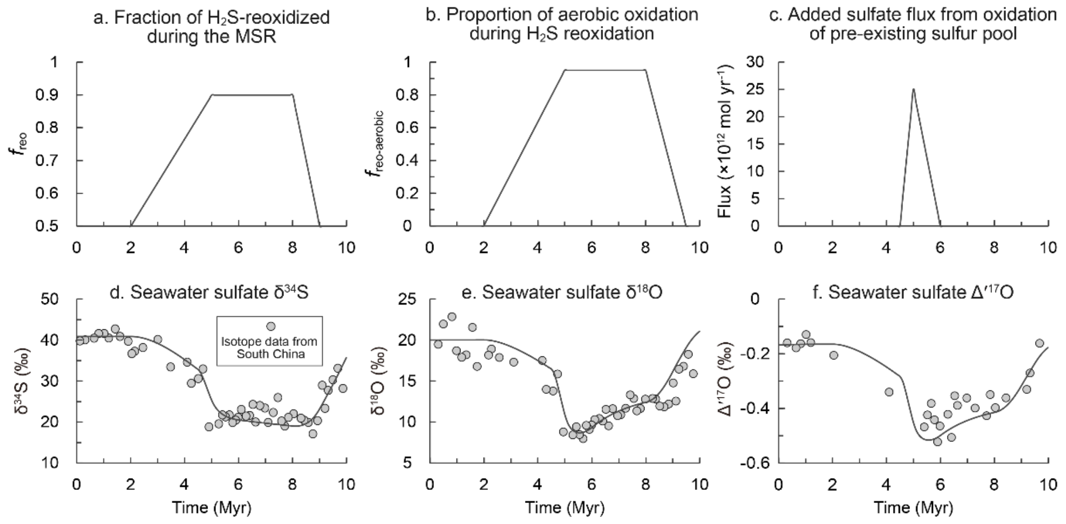

Supplementary Fig. 5. Model output when initial steady-state is SS14-2. Driven forces: changes in the fraction of H_2S-reoxidized (f_{reo}) during the MSR (**a**), proportion of aerobic oxidation during H_2S reoxidation ($f_{reo\text{-}aerobic}$) (**b**), and added sulfate flux from oxidation of the pre-existing sulfur pool (**c**). Model results: seawater sulfate $\delta^{34}S$ (**d**), $\delta^{18}O$ (**e**), and $\Delta'^{17}O$ (**f**).

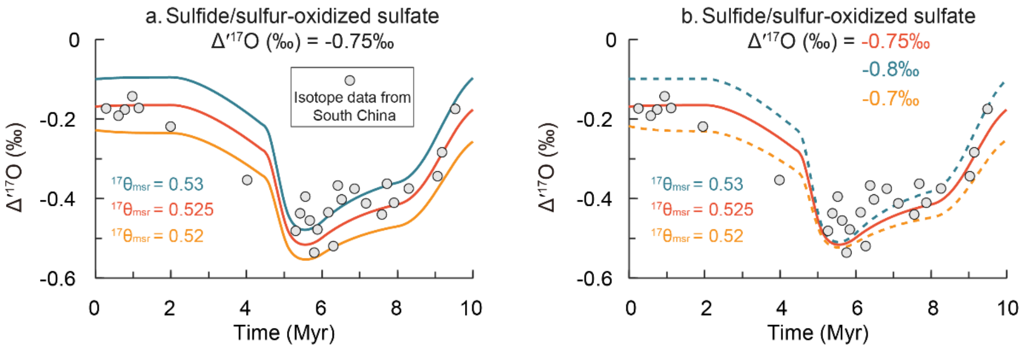

Supplementary Fig. 6. Sensitivity tests of sulfate $\Delta'^{17}O$ on the choice of $^{17}\theta_{msr}$. (**a**) Changing $^{17}\theta_{msr}$ from 0.52 to 0.53 while keeping the $\Delta'^{17}O_{sul\text{-}oxidized}$ value at −0.75‰, (**b**) Changing $^{17}\theta_{msr}$ from 0.52 to 0.53 while allowing the $\Delta'^{17}O_{sul\text{-}oxidized}$ value to vary to fit the observed most negative sulfate $\Delta'^{17}O$ value at ~ −0.5‰ during the SE.

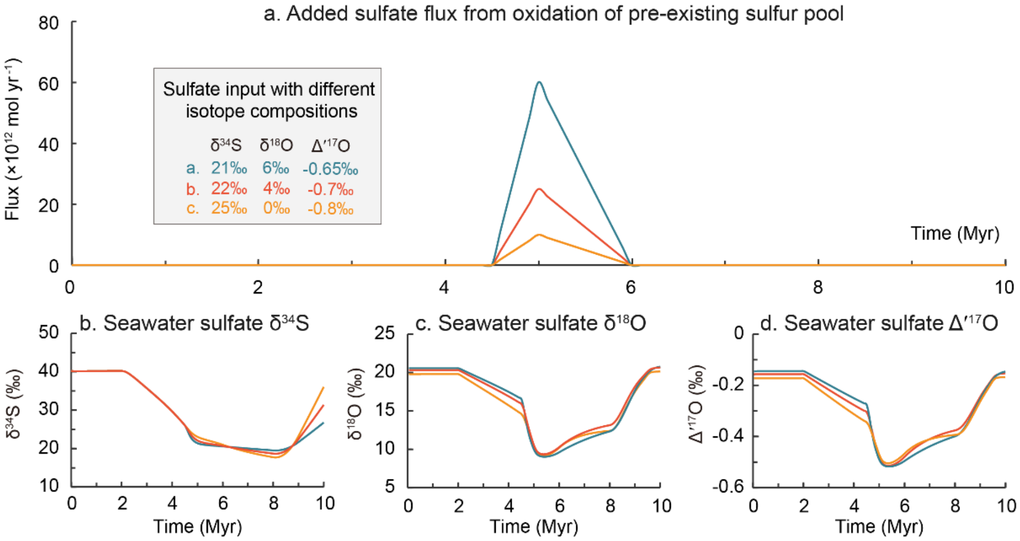

Supplementary Fig. 7. Sensitivity tests of the flux of the added sulfate on the choice of the isotope composition of the added sulfate (**a**) while fitting the observed temporal trends of sulfate $\delta^{34}S$ (**b**), $\delta^{18}O$ (**c**), and $\Delta'^{17}O$ (**d**).

Supplementary Tables

Supplementary Table 1. Isotope data comparison between SE versus pre- and post-SE samples.

		Pre-SE			SE*			Post-SE		
		South China	South Australia	Tarim	South China	South Australia	Tarim	South China	South Australia	Tarim
$\delta^{34}S_{CAS}$ ‰ V-CDT	Min	32.7	-	11.0	16.3	15.2	10.9	-	17.1	10.2
	Max	42.0	-	14.9	33.9	21.4	18.4	-	29.1	13.8
	Mean	38.8	-	12.3	22.8	18.8	13.8	-	23.0	12.1
	Median	39.4	-	11.6	20.9	19.4	13.4	-	22.4	12.7
$\delta^{18}O_{CAS}$ ‰ V-SMOW	Min	16.9	-	13.1	8.0	8.8	10.4	-	12.6	12.3
	Max	23.1	-	16.1	18.4	14.8	15.0	-	15.3	13.4
	Mean	19.3	-	14.2	12.2	12.2	12.4	-	13.9	12.8
	Median	18.6	-	13.9	11.7	12.6	12.2	-	14.1	12.8
$\Delta'^{17}O_{CAS}$ ‰	Min	-0.21	-	-0.20	-0.53	-0.51	-0.35	-	-0.24	-0.10
	Max	-0.14	-	0.06	-0.17	-0.14	-0.05	-	-0.07	-0.03
	Mean	-0.17	-	-0.02	-0.40	-0.26	-0.17	-	-0.19	-0.05
	Median	-0.17	-	0.03	-0.40	-0.23	-0.15	-	-0.20	-0.04

*Note that we arbitrarily set the SE interval to be where the $\delta^{13}C_{carb}$ values are lower than −6‰.

Supplementary Table 2. Fluxes, isotope compositions, fractionation factors, and associated parameters for model.

Parameter	Initial values	^{34}S ‰ (V-CDT)	^{18}O ‰ (V-SMOW)	Source
V_{SO4}, ocean volume	1.38×10^{21} L			10,22#
F_{wp}, pyrite weathering flux	1.3×10^{12} mol yr^{-1}	−17*	0	10,22,32#
F_{we}, evaporite weathering flux	1.5×10^{12} mol yr^{-1}	22	13	10,22,32#
F_{v}, mantle degassing flux	0.5×10^{12} mol yr^{-1}	0	3	10,22
$F_{MSR\text{-}r}$, intracellular backward flux			25	25–27
$F_{reo\text{-}aerobic}$, aerobic sulfide/sulfur oxidation			4†	29–31
$F_{reo\text{-}anaerobic}$, anaerobic sulfide/sulfur oxidation			21	28
k_{msr}, coefficient of MSR flux in the water column	0-10			
f_{reo}, fraction of H_2S-reoxidized during MSR	0.5-0.9			
$f_{reo\text{-}aerobic}$, proportion of aerobic H_2S oxidation	0-0.3			
$\Delta'^{17}O_{sul\text{-}oxidized}$, sulfate from sulfide/sulfur oxidation	−0.7‰ to −0.85‰			
$\alpha^{34}S_{MSR}$, sulfur isotope fractionation during MSR	0.961-0.964			
$^{17}\theta_{msr}$, mass law of MSR	0.52-0.53			

*$\delta^{34}S_{py}$ value is set to −17‰ in the typical model, but a value of 18‰ is also considered in one case. See Supplementary Fig. 4b.

†The $\delta^{18}O$ of sulfate from aerobic sulfur oxidation is set to 4‰ in the typical model, and a range of 0 to 6‰ was also explored. See Supplementary Fig. 7.

#Also see the references therein.

Supplementary Table 3. Initial steady states for marine sulfur cycling during the pre-SE time.

Parameter	$\delta^{34}S$ ‰	$\delta^{18}O$ ‰	$\Delta'^{17}O$ ‰	k_{msr}	F_{MSR} $\times 10^{13}$ mol yr^{-1}	r_{msr}	f_{reo}	$f_{reo\text{-}aerobic}$	$^{18}\alpha_{MSR}$	$\Delta'^{17}O_{sul\text{-}oxidized}$ ‰	$^{17}\theta_{MSR}$	$[SO_4]$ mM
SS1	40.7	20.4	–0.06	10	1.77	0.54	0.6	0	0.964	0	0.528	0.2
SS2	40.6	20.4	–0.06	8	1.77	0.54	0.6	0	0.964	0	0.528	0.2
SS3	40.6	20.4	–0.06	6	1.77	0.54	0.6	0	0.964	0	0.528	0.3
SS4-1	40.5	20.4	–0.06	4	1.77	0.54	0.6	0	0.964	0	0.528	0.5
SS4-2	40.5	20.4	–0.15	4	1.77	0.54	0.6	0	0.964	–0.7	0.525	0.5
SS5	40.3	20.6	–0.06	5	3.55	0.54	0.8	0.2	0.964	0	0.528	1.0
SS6	40.3	20.4	–0.06	2	1.77	0.54	0.6	0	0.964	0	0.528	1.0
SS7	40.3	20.1	–0.06	7	4.68	0.54	0.85	0.3	0.964	0	0.528	1.0
SS8-1	40.2	20.3	–0.06	10	7.00	0.53	0.9	0.3	0.964	0	0.528	1.1
SS8-2	40.2	20.3	–0.15	10	7.00	0.53	0.9	0.3	0.964	–0.7	0.528	1.1
SS9-1	40	20.3	–0.06	8	6.96	0.53	0.9	0.3	0.964	0	0.528	1.5
SS9-2	40	20.3	–0.1	8	6.96	0.53	0.9	0.3	0.964	0	0.525	1.5
SS9-3	40	20.3	–0.03	8	6.96	0.53	0.9	0.3	0.964	0	0.53	1.5
SS9-4	40	20.3	–0.17	8	6.96	0.53	0.9	0.3	0.964	–0.8	0.528	1.5
SS10-1	40	20.1	–0.06	5	4.64	0.53	0.85	0.3	0.964	0	0.528	1.5
SS10-2	40	20.1	–0.17	5	4.64	0.53	0.85	0.3	0.964	–0.8	0.528	1.5
SS11-1	40.8	20.1	–0.06	2	2.86	0.55	0.75	0.25	0.963	0	0.528	2.2
SS11-2	40.8	20.1	–0.17	2	2.86	0.55	0.75	0.25	0.963	–0.85	0.528	2.2
SS12	40.6	20.5	–0.06	6	7.11	0.55	0.9	0.3	0.963	0	0.528	2.5
SS13	40.1	20.2	–0.06	3	4.68	0.55	0.85	0.3	0.963	0	0.528	3.6
SS14-1	40.9	20	–0.06	0	1.46	0.58	0.5	0	0.961	0	0.528	6.0
SS14-2	40.9	20	–0.17	0	1.46	0.58	0.5	0	0.961	–0.75	0.525	6.0
SS14-3	40.9	20	–0.17	0	1.46	0.58	0.5	0	0.961	0	0.52	6.0

Supplementary References

1. Lu, M. *et al.* The DOUNCE event at the top of the Ediacaran Doushantuo Formation, South China: Broad stratigraphic occurrence and non-diagenetic origin. *Precambrian Res* **225**, 86–109 (2013).
2. Husson, J. M., Maloof, A. C., Schoene, B., Chen, C. Y. & Higgins, J. A. Stratigraphic expression of earth's deepest $\delta^{13}C$ excursion in the Wonoka formation of South Australia. *Am J Sci* **315**, 1–45 (2015).
3. McFadden, K. A. *et al.* Pulsed oxidation and biological evolution in the Ediacaran Doushantuo Formation. *Proc Natl Acad Sci* **105**, 3197–3202 (2008).
4. Tahata, M. *et al.* Carbon and oxygen isotope chemostratigraphies of the Yangtze platform, South China: Decoding temperature and environmental changes through the Ediacaran. *Gondwana Research* **23**, 333–353 (2013).
5. Xu, B. *et al.* SHRIMP zircon U–Pb age constraints on Neoproterozoic Quruqtagh diamictites in NW China. *Precambrian Res* **168**, 247–258 (2009).
6. Ren, R., Guan, S. W., Zhang, S. C., Wu, L. & Zhang, H. Y. How did the peripheral subduction drive the Rodinia breakup: Constraints from the Neoproterozoic tectonic process in the northern Tarim Craton. *Precambrian Res* **339**, 105612 (2020).
7. Xiao, S. *et al.* The Neoproterozoic Quruqtagh Group in eastern Chinese Tianshan: Evidence for a post-Marinoan glaciation. *Precambrian Res* **130**, 1–26 (2004).
8. Wang, Y., Chen, D., Liu, M., Liu, K. & Tang, P. Ediacaran carbon cycling and Shuram excursion recorded in the Tarim. *Precambrian Res* **377**, 106694 (2022).
9. Dodd, M. S. *et al.* Uncovering the Ediacaran phosphorus cycle. *Nature* (2023) doi:10.1038/s41586-023-06077-6.
10. Yao, W., Paytan, A. & Wortmann, U. G. Effects of a transient marine sulfur

reservoir on seawater $\delta^{18}O_{SO4}$ during the Paleocene-Eocene Thermal Maximum. *Geochim Cosmochim Acta* **269**, 257–269 (2020).

11. Gomes, M. L. & Johnston, D. T. Oxygen and sulfur isotopes in sulfate in modern euxinic systems with implications for evaluating the extent of euxinia in ancient oceans. *Geochim Cosmochim Acta* **205**, 331–359 (2017).
12. Wing, B. A. & Halevy, I. Intracellular metabolite levels shape sulfur isotope fractionation during microbial sulfate respiration. *Proc Natl Acad Sci* **111**, 18116–18125 (2014).
13. Jørgensen, B. B. Sulfur Biogeochemical Cycle of Marine Sediments. *Geochem Perspect* **10**, 145–307 (2021).
14. Cao, X. & Bao, H. Small Triple Oxygen Isotope Variations in Sulfate: Mechanisms and Applications. *Rev Mineral Geochem* **86**, 463–488 (2021).
15. Waldeck, A. R. *et al.* Deciphering the atmospheric signal in marine sulfate oxygen isotope composition. *Earth Planet Sci Lett* **522**, 12–19 (2019).
16. Wortmann, U. G. & Chernyavsky, B. M. Effect of evaporite deposition on Early Cretaceous carbon and sulphur cycling. *Nature* **446**, 654–656 (2007).
17. Jørgensen, B. B. The sulfur cycle of a coastal marine sediment (Limfjorden, Denmark)1. *Limnol Oceanogr* **22**, 814–832 (1977).
18. Jørgensen, B. B. Mineralization of organic matter in the sea bed—the role of sulphate reduction. *Nature* **296**, 643–645 (1982).
19. Turchyn, A. V. & Schrag, D. P. Oxygen Isotope Constraints on the Sulfur Cycle over the Past 10 Million Years. *Science* **303**, 2004–2007 (2004).
20. Brunner, B. & Bernasconi, S. M. A revised isotope fractionation model for dissimilatory sulfate reduction in sulfate reducing bacteria. *Geochim Cosmochim Acta* **69**, 4759–4771 (2005).

21. Brüchert, V. *et al.* Regulation of bacterial sulfate reduction and hydrogen sulfide fluxes in the central Namibian coastal upwelling zone. *Geochim Cosmochim Acta* **67**, 4505–4518 (2003).

22. Wortmann, U. G. & Paytan, A. Rapid Variability of Seawater Chemistry Over the Past 130 Million Years. *Science* **337**, 334–336 (2012).

23. Oba, Y. & Poulson, S. R. Oxygen isotope fractionation of dissolved oxygen during abiological reduction by aqueous sulfide. *Chem Geol* **268**, 226–232 (2009).

24. Chen, K. Y. & Morris, J. C. Kinetics of oxidation of aqueous sulfide by oxygen. *Environ Sci Technol* **6**, 529–537 (1972).

25. Zeebe, R. E. A new value for the stable oxygen isotope fractionation between dissolved sulfate ion and water. *Geochim Cosmochim Acta* **74**, 818–828 (2010).

26. Fritz, P., Basharmal, G. M., Drimmie, R. J., Ibsen, J. & Qureshi, R. M. Oxygen isotope exchange between sulphate and water during bacterial reduction of sulphate. *Chemical Geology: Isotope Geoscience section* **79**, 99–105 (1989).

27. Farquhar, J., Canfield, D. E., Masterson, A., Bao, H. & Johnston, D. Sulfur and oxygen isotope study of sulfate reduction in experiments with natural populations from Fællestrand, Denmark. *Geochim Cosmochim Acta* **72**, 2805–2821 (2008).

28. Böttcher, M. E., Thamdrup, B., Gehre, M. & Theune, A. $^{34}S/^{32}S$ and $^{18}O/^{16}O$ fractionation during sulfur disproportionation by Desulfobulbus propionicus. *Geomicrobiol J* **22**, 219–226 (2005).

29. Balci, N., Shanks, W. C., Mayer, B. & Mandernack, K. W. Oxygen and sulfur isotope systematics of sulfate produced by bacterial and abiotic oxidation of pyrite. *Geochim Cosmochim Acta* **71**, 3796–3811 (2007).

30. Balci, N., Mayer, B., Shanks, W. C. & Mandernack, K. W. Oxygen and sulfur isotope systematics of sulfate produced during abiotic and bacterial oxidation of sphalerite and elemental sulfur. *Geochim Cosmochim Acta* **77**, 335–351 (2012).

31. Van Stempvoort, D. R. & Krouse, H. R. Controls of $\delta^{18}O$ in sulfate-Review of experimental data and application to specific environments in *Environmental Geochemistry of Sulfide Oxidation,* C. N. Alpers, D. W. Blowes Eds. American Chemical Society, Washington, D.C., pp. 446–480 (1994).

32. Burke, A. *et al.* Sulfur isotopes in rivers: Insights into global weathering budgets, pyrite oxidation, and the modern sulfur cycle. *Earth Planet Sci Lett* **496**, 168–177 (2018).

33. Yao, W., Paytan, A. & Wortmann, U. G. Large-scale ocean deoxygenation during the Paleocene-Eocene Thermal Maximum. *Science* **361**, 804–806 (2018).

34. Crockford, P. W. *et al.* Claypool continued: Extending the isotopic record of sedimentary sulfate. *Chem Geol* **513**, 200–225 (2019).

35. Crockford, P. W. *et al.* Triple oxygen isotope evidence for limited mid-Proterozoic primary productivity. *Nature* **559**, 613–616 (2018).
