# Supplementary material for: Sulfate triple-oxygen-isotope evidence confirming oceanic oxygenation 570 million years ago
Source: Nat Commun. 2023 Jul 18;14:4315. doi: 10.1038/s41467-023-39962-9 (PMC10354052; doi:10.1038/s41467-023-39962-9)
Supplement: Supplementary file 3 — Description of Additional Supplementary Files [file 41467_2023_39962_MOESM3_ESM.pdf]

Description of Additional Supplementary Files:

Supplementary Data 1: Isotope data from three different paleocontinents measured in this study.
